# Angioblast Derived from ES Cells Construct Blood Vessels and Ameliorate Diabetic Polyneuropathy in Mice

**DOI:** 10.1155/2015/257230

**Published:** 2015-04-21

**Authors:** Tatsuhito Himeno, Hideki Kamiya, Keiko Naruse, Zhao Cheng, Sachiko Ito, Taiga Shibata, Masaki Kondo, Jiro Kato, Tetsuji Okawa, Atsushi Fujiya, Hirohiko Suzuki, Tetsutaro Kito, Yoji Hamada, Yutaka Oiso, Kenichi Isobe, Jiro Nakamura

**Affiliations:** ^1^Department of Endocrinology and Diabetes, Nagoya University Graduate School of Medicine, 65 Tsurumai-cho, Showa-ku, Nagoya 466-8550, Japan; ^2^Department of Immunology, Nagoya University Graduate School of Medicine, 65 Tsurumai-cho, Showa-ku, Nagoya 466-8550, Japan; ^3^Department of Chronic Kidney Disease Initiatives, Nagoya University Graduate School of Medicine, 65 Tsurumai-cho, Showa-ku, Nagoya 466-8550, Japan; ^4^Division of Diabetes, Department of Internal Medicine, Aichi Medical University School of Medicine, 21 Karimata, Yazako, Nagakute, Aichi 480-1195, Japan; ^5^Department of Internal Medicine, School of Dentistry, Aichi Gakuin University, 1-100 Kusumoto-cho, Chikusa-ku, Nagoya 464-8650, Japan; ^6^Department of Metabolic Medicine, Nagoya University Graduate School of Medicine, 65 Tsurumai-cho, Showa-ku, Nagoya 466-8550, Japan

## Abstract

*Background*. Although numerous reports addressing pathological involvements of diabetic polyneuropathy have been conducted, a universally effective treatment of diabetic polyneuropathy has not yet been established. Recently, regenerative medicine studies in diabetic polyneuropathy using somatic stem/progenitor cell have been reported. However, the effectiveness of these cell transplantations was restricted because of their functional and numerical impairment in diabetic objects. Here, we investigated the efficacy of treatment for diabetic polyneuropathy using angioblast-like cells derived from mouse embryonic stem cells. *Methods and Results*. Angioblast-like cells were obtained from mouse embryonic stem cells and transplantation of these cells improved several physiological impairments in diabetic polyneuropathy: hypoalgesia, delayed nerve conduction velocities, and reduced blood flow in sciatic nerve and plantar skin. Furthermore, pathologically, the capillary number to muscle fiber ratios were increased in skeletal muscles of transplanted hindlimbs, and intraepidermal nerve fiber densities were ameliorated in transplanted plantar skin. Transplanted cells maintained their viabilities and differentiated to endothelial cells and smooth muscle cells around the injection sites. Moreover, several transplanted cells constructed chimeric blood vessels with recipient cells. *Conclusions*. These results suggest that transplantation of angioblast like cells induced from embryonic stem cells appears to be a novel therapeutic strategy for diabetic polyneuropathy.

## 1. Introduction

Large prospective clinical studies have shown a strong relationship between hyperglycemia and diabetic microvascular complications in both type 1 diabetes and type 2 diabetes [1–3]. Several hypotheses about diabetic complications have been formulated: increased polyol pathway flux [4], increased advanced glycation end-product formation [5–10], increased PKC activation [11–14], and increased hexosamine pathway flux [15]. The involvement of these chronic hyperglycemia-mediated metabolic aberrances has also been proven in diabetic polyneuropathy (DPN) [16, 17].

DPN is the most common peripheral neuropathy, and degeneration of distal axons of peripheral neurons progresses slowly and symmetrically in DPN. Along with HbA1c and duration of diabetes, baseline cardiovascular disease and smoking have been indicated as risk factors for DPN [18]. Additionally, it has been reported that, in DPN, the thickness of endoneurial microvessels of sural nerves increased [19, 20] while endoneurial nutritive blood flow of sciatic nerves decreased [21, 22]. The vascular dysfunction in DPN is considered to be one of the most important pathologies.

Evidence accumulated in the past two decades indicates that peripheral blood cells contain a subpopulation with properties similar to embryonic angioblast [23–26]. These cells could be differentiated into mature endothelial cells. Therefore, these cells were termed “endothelial progenitor cells” (EPCs). It has been attested that EPCs migrated to ischemia lesions and played a pivotal role in vascular endothelial function and angiogenesis [27, 28]. Several studies have demonstrated the interventional effects of EPC transplantation on myocardial ischemia/infarction and preventive effects on plaque progression in mice [29, 30]. However, the number of circulating EPCs decreased in diabetic patients, and EPCs isolated from diabetic mice exhibited decreased functions, including migration, adhesion, and tube formation [31–35]. Recent studies have shown that oxidative stress, elevated by hyperglycemia, decreased EPC survival through inhibition of cell proliferation and NO production [36, 37]. Marrotte et al. reported that EPCs isolated from diabetic mice were less effective than EPCs from nondiabetic mice at accelerating wound closure in diabetic mice [38]. They also demonstrated that decreased expression of manganese superoxide dismutase in EPCs obtained from diabetic mice contributes to impairment of wound healing. These functional and metabolic obstacles inhibit clinical applications of EPCs on diabetic vascular complications.

In current regenerative medicines, cells used for transplantations are expected to have two primary abilities: one is differentiation abilities to specific cells or tissues and the other is paracrine supportive effects to impaired tissues. Certain cell transplantation therapies have already been applied to some clinical diseases using various stem/progenitor cells from somatic tissues: EPC, mesenchymal stem cell (MSC), skin-derived precursor cell, neural stem cell, and neural crest stem cell [39, 40]. In particular, MSC has been applied to the treatment of certain diseases: myocardial infarction [41], stroke [42], and graft versus host disease [43]. Although the paracrine mechanisms in damaged tissues and the immunomodulatory properties of MSC have been proven in these clinical applications, the probability of survival of transplanted MSCs is small and the therapeutic effects on a long-term basis remain unclear.

Recent advances of stem cell biology permit the derivation of induced pluripotent stem (iPS) cell lines at an individual level in mammalians including humans. Although ES cells are attractive in their pluripotency and availability of intact and stable undifferentiated status, there are difficulties in clinical applications of human ES cells because of safety and ethics. Although there are still safety problems that have to be solved, the autologous use of human iPS cells may resolve ethical issues. In this circumstance, problematic issues with using ES cells could be circumvented by employing an equivalent substance, iPS cells, in the future. However, there is no standard for iPS cells: the number of obtainable iPS cell lines is vast and the methods to obtain them are innumerable. Additionally, there are heterogeneous properties among the iPS cells. Therefore, we utilized ES cells in this current study.

We have reported that transplantation of EPCs ameliorated DPN [44]. We hypothesized that if angioblast-like cells could be obtained from iPS or ES cells, these cells would operate like EPCs and restore numerous impairments in DPN. It has been shown that the vascular endothelial growth factor receptor-2 (VEGFR2, known also Flk1) was expressed in angioblasts and its function is essential for the differentiation of endothelial cells and hematopoietic cells [45]. Therefore, Flk1 positive (Flk1^+^) cells derived from ES cells may have similar characteristics to angioblasts and similar or additional functions of EPCs in adults. This is the first report that demonstrates the therapeutic effects of transplantation of angioblast-like cells derived from mouse ES cells on DPN.

## 2. Research Design and Methods

### 2.1. Cell Culture

BRC6, mouse ES cells derived from C57BL/6 female embryo, and B6G02, mouse ES cells expressing GFP derived from a C57BL/6 male embryo, were obtained from the Riken Cell Bank (Ibaragi, Japan). ES cells were maintained in DMEM (Invitrogen, Van Allen Way Carlsbad, CA) containing 10% Knockout Serum Replacement (Invitrogen), 1% FBS (Sigma-Aldrich, St. Louis, MO), nonessential amino acids (Invitrogen), 5.5 mmol/L 2-mercaptoethanol (GIBCO, Burlington, ON), 50 U/mL penicillin (GIBCO), and 50 mg/mL streptomycin (GIBCO) on feeder layers of mitomycin C-inactivated SNL76/7 cells (the European Collection of Cell Cultures, Salisbury, UK), which were clonally derived from an STO cell line transfected with a G418 resistance cassette and a leukemia inhibitory factor expression construct [46]. The STO cells were established from fibroblasts derived from male and female embryos.

Cell differentiation was induced as previously described [47]. In brief, a differentiation medium (*α*-MEM (Invitrogen) supplemented with 10% FBS and 5 × 10^−5 ^mol/L 2-mercaptoethanol) was used for ES cell differentiation. For the induction, ES cells were plated at 1.7 × 10^3^ cells/cm^2^ in the differentiation medium on gelatin-coated dishes, which were coated with 0.1% gelatin (Sigma-Aldrich) solution and cultured for 4 days.

Immortalized Schwann cells (IMS32), established by a long-term culture of adult mouse DRGs and peripheral nerves [48], were a kind gift from Dr. Watabe. IMS32s were cultured in DMEM (Sigma-Aldrich) containing 5.5 mM D-glucose, penicillin- (100 U/mL) streptomycin (100 mg/mL), and 5% FBS (Moregate Biotech, Bulimba QLD, Australia). PA6 cells, a type of mouse MSC, were obtained from the Riken Cell Bank. The PA6 cells were cultured in *α*-MEM supplemented with 10% FBS and 5 × 10^−5 ^mol/L 2-mercaptoethanol.

### 2.2. Cell Sorting

Differentiated cells were detached using Accutase (Sigma-Aldrich) after a 4-day culture. Dissociated cells were reacted with Allophycocyanin conjugated anti-mouse Flk1 antibody (eBioscience, San Diego, CA), followed by sorting with a magnetic cell separation system (MACS) (Miltenyi Biotec, Bergisch Gladbach, Germany). The purity of the sorted cell population was confirmed in a fluorescence-activated cell sorting (FACS) analysis (BD FACS Canto, BD, Franklin Lakes, NJ). A portion of the separated cells was transferred to RNAlater Solution (Invitrogen) followed by a freezing preservation for reverse-transcription PCR (RT-PCR).

### 2.3. In Vitro Induction of Endothelial Cell and Smooth Muscle Cell

Sorted Flk1^+^ cells were plated on gelatin-coated dishes in the differentiation medium. For induction to an endothelial cell, differentiation medium supplemented with 100 ng/mL human VEGF_165_ (R&D Systems, Minneapolis, MN) was used. The medium was replaced every 2 days. After a 4-day culture, cells were fixed with 4% PFA (Wako Pure Chemical, Osaka, Japan) for 20 minutes at 4°C.

Then, specimens were blocked with 3% goat serum (Vector Laboratories, Burlingame, CA) with PBS (GIBCO) for 30 minutes at room temperature, and the following primary antibodies were applied to the sections at 4°C overnight: rabbit polyclonal anti-*α*-SMA antibody (1 : 200; Santa Cruz Biotechnology Inc., Santa Cruz, CA) or rabbit polyclonal anti-PECAM antibody (1 : 200; Santa Cruz Biotechnology Inc.). After washing, the following secondary antibodies were loaded for 1 hour at room temperature in a dark box: Alexa Fluor 488-coupled goat anti-rabbit IgG antibody (1 : 200; Invitrogen) or Alexa Fluor 594-coupled goat anti-rabbit antibody (1 : 300; Invitrogen). Finally, nucleus staining was performed using DAPI (Merck, Tokyo, Japan). The stained sections were observed using a fluorescence microscope (BX51, Olympus Optical, Tokyo, Japan) and images were obtained by a CCD camera (DP70, Olympus Optical).

### 2.4. Tube Formation Assay

For the tube formation assay, the cells separated by MACS were plated on 4-well slides (Iwaki Glass, Tokyo, Japan) coated thickly with BD Matrigel Basement Membrane Matrix (250 *μ*L, Becton-Dickinson Co., Franklin Lakes, NJ) at 3 × 10^4^ cells/cm^2^, following culture for 48 hours in EBM-2 medium with EGM-2 Bulletkit (LONZA, Basel, Switzerland). After the culture, cells were fixed with 4% PFA and stained with Alexa Fluor 594 conjugated isolectin GS-IB4 (Invitrogen) and DAPI (Merck). The tube formations were assessed with a fluorescence microscope (BX51, Olympus Optical) and images were obtained by a CCD camera (DP70, Olympus Optical).

### 2.5. Animals and Induction of Diabetes

Five-week-old male C57BL/6 mice (Chubu Kagaku Shizai, Nagoya, Japan) with an initial body weight of 24–26 g were allowed to adapt to the experimental animal facility for 7 days. The animals were housed in an aseptic animal room under controlled light/dark and temperature conditions with food and water available ad libitum. Diabetes was induced by intraperitoneal injection (i.p.) of streptozotocin (STZ) (150 mg/kg; Sigma-Aldrich). Control mice received an equal volume of citric acid buffer (Wako). One week after STZ administration, the mice with plasma glucose concentrations of >16 mmol/L were defined as diabetic mice. Twelve weeks after the induction of diabetes, 1 × 10^5^ cells/limb of purified Flk1^+^ cells in 0.2 mL saline were injected into the right thigh and soleus muscles of both the normal and the diabetic mice. The left hindlimb muscles were treated with saline alone. Four weeks later, the following parameters were bilaterally measured. Before transplantation of Flk1^+^ cells, fasting blood glucose levels and hemoglobin A1c were examined by a FreeStyle Freedom Glucose Meter (Nipro, Osaka, Japan) and a RAPIDIA Auto HbA1c-L assay kit using latex agglutination (Fujirebio Inc., Tokyo, Japan), respectively. The Nagoya University Institutional Animal Care and Use Committee approved the protocols of this experiment.

### 2.6. Measurement of Current Perception Thresholds (CPTs) Using a Neurometer

To determine a nociceptive threshold, CPTs were measured in 12- and 16-week diabetic and age-matched normal mice using a CPT/LAB Neurometer (Neurotron, Denver, CO), according to the method by Shibata et al. [49] with minor modifications. The electrodes (SRE-0405-8; Neurotron) for stimulation were attached to plantar surfaces of the mice. Each mouse was kept in a Ballman cage (Natsume Seisakusho, Tokyo, Japan) suitable for light restraint to keep the mice awake. Three transcutaneous-sine-wave stimuli with different frequencies (2000, 250, and 5 Hz) were applied to the plantar surfaces of the mice to determine the CPT of sensory perceptions (pressure, pain, and pain and temperature, resp.). The intensity of each stimulation was automatically increased in gradual increments (0.01 mA for 5 and 250 Hz and 0.02 mA for 2000 Hz). The minimum intensity at which each mouse withdrew its paw was defined as the CPT. Six consecutive measurements were conducted at each frequency.

### 2.7. Nerve Conduction Velocities (NCVs)

After intraperitoneal injection of sodium pentobarbital (5 mg/100 g, Kyoritsuseiyaku Corporation, Tokyo, Japan), mice were placed on a heated pad in a room maintained at 25°C to ensure a constant rectal temperature of 37°C. Motor nerve conduction velocity (MNCV) was determined between the ankle and sciatic notch using a Neuropak NEM-3102 instrument (Nihon-Koden, Osaka, Japan), as previously described [42, 49, 50]. The sensory nerve conduction velocity (SNCV) was measured between the knee and ankle with retrograde stimulation.

### 2.8. Blood Flows of Sciatic Nerve and Plantar Skin

After evaluation of the MNCV and SNCV, blood flows of the sciatic nerve and the plantar skin were measured by laser Doppler flowmetry (FLO-N1; Omegawave Inc, Tokyo, Japan). To determine sciatic nerve blood flow, the thigh skin of an anesthetized mouse was cut along femur and then an incision through the fascia was carefully performed to expose the sciatic nerve. Five minutes after this procedure, the blood flow was measured by a laser Doppler probe placed 1 mm above the nerve. The skin blood flow was determined by the mean of the flow at 3 spots. During this measurement, the mouse was placed on a heated pad in a room maintained at 25°C to ensure a constant rectal temperature of 37°C.

### 2.9. Tissue Collection

Four weeks after the transplantation, mice were sacrificed by an overdose of pentobarbital (10 mg/100 g). Soleus muscles and plantar skin tissue were obtained from normal and diabetic mice. Some tissues were snap-frozen in liquid nitrogen followed by preservation at −80°C until use, and others were transferred to RNAlater Solution (Invitrogen) followed by freezing preservation for RT-PCR.

### 2.10. Real-Time RT-PCR

RNA was extracted from frozen samples of cells and tissues using ISOGEN (Nippon Gene, Toyama, Japan) according to the manufacturer's instructions and the concentrations were quantified spectrophotometrically (NanoDrop ND-100, Nanodrop Tec., Wilmington, DE). DNA was digested with RNase-free DNase I (Wako Pure Chemical), which was then inactivated by incubation at 80°C for 10 min.

Starting from 1 *μ*g of RNA, cDNA was synthesized using ReverTra Ace (TOYOBO, Osaka, Japan) according to the manufacturer's descriptions. Primers were designed by Primer3 software (http://frodo.wi.mit.edu/) and tested for specificity with NCBI-BLAST (http://www.ncbi.nlm.nih.gov/tools/primer-blast/). Real-time quantitative RT-PCR was performed and monitored using Mx3000P QPCR System (STRATAGENE, La Jolla, California, USA). The PCR master mix was based on SYBR Green PCR Master Mix (Applied Biosystems, Foster City, CA). In reaction wells, cDNA samples (5 *μ*L for a total volume of 25 *μ*L per reaction) were analyzed for the gene of interest. All data were normalized to an internal standard, *β*-actin. The PCR products were analyzed by agarose gel (Takara Bio Inc., Otsu, Japan)/ethidium bromide (Sigma-Aldrich) to confirm these predicted lengths. Relative quantity was calculated by the ΔΔCt method. The primer sequences are shown in Table 1.

### 2.11. Immunohistochemistry

Four weeks after the transplantation, the mice were anesthetized with sodium pentobarbital (5 mg/100 g) and perfused with 50 mL of 4% PFA fixative. After perfusion, soleus muscles and plantar skin samples were excised and fixed in 4% PFA at 4°C overnight. Specimens were immersed in PBS containing 20% sucrose (Wako) embedded within an O.C.T. compound (Sakura Finetechnical, Tokyo, Japan) and cut into 5 *μ*m sections with a sliding cryostat (CM1800, Leica Microsystems AG, Wetzler, Germany). After 5 minutes of microwave irradiation in citrate buffer (pH 6.0, Wako), the cryostat sections were blocked with 3% goat serum with PBS for 30 minutes at room temperature. Prior to being stored at 4°C overnight, the following primary antibodies were applied to the sections: rabbit polyclonal anti-protein-gene-product 9.5 (PGP 9.5) antibody (1 : 500; Millipore, Billerica, MA), rabbit polyclonal anti-*α*-SMA antibody (1 : 200; Santa Cruz Biotechnology Inc.), and anti-rabbit PECAM antibody (1 : 200; Santa Cruz Biotechnology Inc.). After the sections were rinsed with PBS, the following secondary antibodies were loaded: Alexa Fluor 594-coupled goat anti-rabbit IgG antibody (1 : 200; Invitrogen). Finally, nucleus staining was performed using DAPI (Merck). The stained sections were observed using a fluorescence microscope (BX51, Olympus Optical) and images were obtained by a CCD camera (DP70, Olympus Optical).

### 2.12. Capillary Number to Muscle Fiber Ratio

Capillary number to muscle fiber ratio was calculated as previously reported [44, 49] with minor modifications. In brief, the sections of soleus muscles fixed with PFA were used for immunostaining. The vascular capillaries were stained by Alexa Fluor 594 conjugate isolectin GS-IB4 (invitrogen) and counted under a fluorescence microscope (BX51, Olympus Optical), and images were obtained by a CCD camera (DP70, Olympus Optical). The muscle fibers were concomitantly counted to determine the capillary number to muscle fiber ratio. Five fields from each section were randomly selected for the capillary counts.

### 2.13. Measurement of Intraepidermal Nerve Fiber Densities (IENFDs)

Nerve fibers stained with anti-PGP 9.5 antibody were counted as previously reported [51]. In brief, the number of nerve fibers was counted at the point of intersection with the basal membrane, regardless of branching within the dermis or epidermis. Six captured images from each section were randomly selected for IENFDs. IENFDs were derived and expressed as epidermal nerve fiber numbers per length of an epidermal basal membrane (fibers/mm).

### 2.14. Statistical Analysis

All of the group values were expressed as means ± SD. Statistical analyses were made by one-way ANOVA, with the Bonferroni correction for multiple comparisons. All analyses were performed by personnel unaware of the animal identities.

## 3. Results

### 3.1. Flk1^+^ Cells Obtained from ES Cells Expressed Mesodermal and Angioblastic Markers

**Figure 1:**
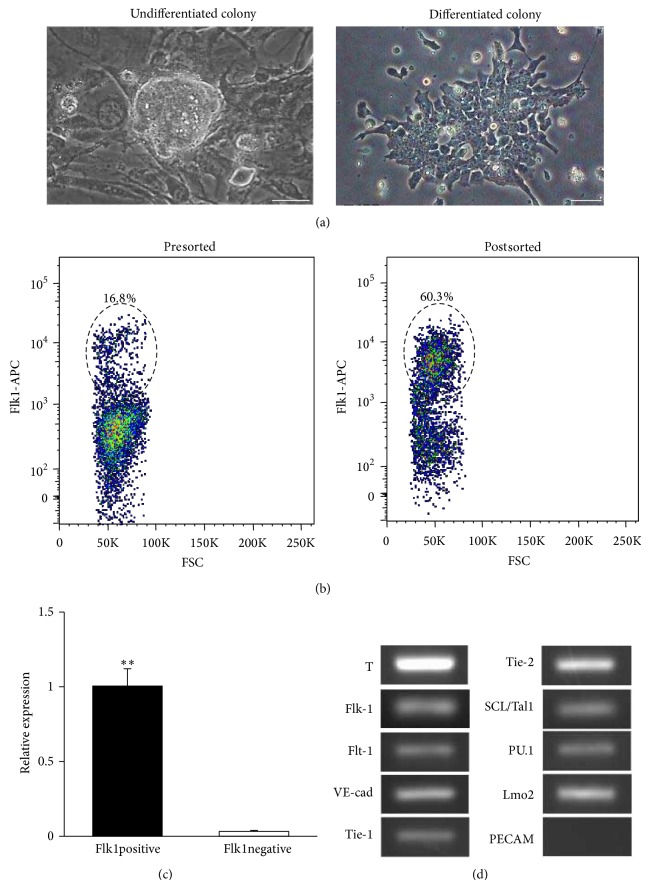
Differentiation and purification of angioblast-like cells from mouse ES cells. (a) Spherical colonies of ES cells (left) were flatly outspread (right) after 4 days of culture on gelatin. Bar: 30 *μ*m. (b) Fluorescence-activated cell sorting analyses showed that a population of cells expressing Flk1 was estimated at 10–20% (left) and came up to 55–63% by magnet associated cell separation (right). (c) Reverse-transcription PCR analyses showed that Flk1 mRNA expression in population selected as Flk1 positive cell was almost 30 times higher than that in population selected as Flk1 negative cell (*P* < 0.001). (d) Reverse-transcribed PCR analyses showed both mesoderm and angioblast markers, that is, Brachyury (T), Flk1, Flt1, Tie1, Tie2, VE-cad, PU.1, SCL/Tal1, and Lmo2, which were expressed in Flk1 positive cells. On the other hand, PECAM, which is considered as a marker of relatively differentiated endothelial cell, was not detected in Flk1 positive cells after the 4 days of differentiation.

After culturing 4 days on gelatin, the shape of the ES cell colonies changed from spherical to planar and the population of cells expressing Flk1 was estimated at 10–20% using FACS analysis (Figures 1(a) and 1(b)). Purification mediated by Flk1 antibody was validated, and Flk1 mRNA expression in Flk1^+^ cells was measured at almost 30 times higher than that in Flk1 negative (Flk1^−^) cells (Figure 1(c)). RT-PCR analyses showed both mesodermal and angioblast markers; that is, Brachyury, Flk1, Flt1, Tie1, Tie2, VE-cad, PU.1, SCL/Tal1, and Lmo2, were expressed in Flk1^+^ cells purified employing MACS (Figure 1(d)).

### 3.2. Flk1^+^ Angioblastic Cells Expressed Angiogenic and Neurotrophic Factors

**Figure 2:**
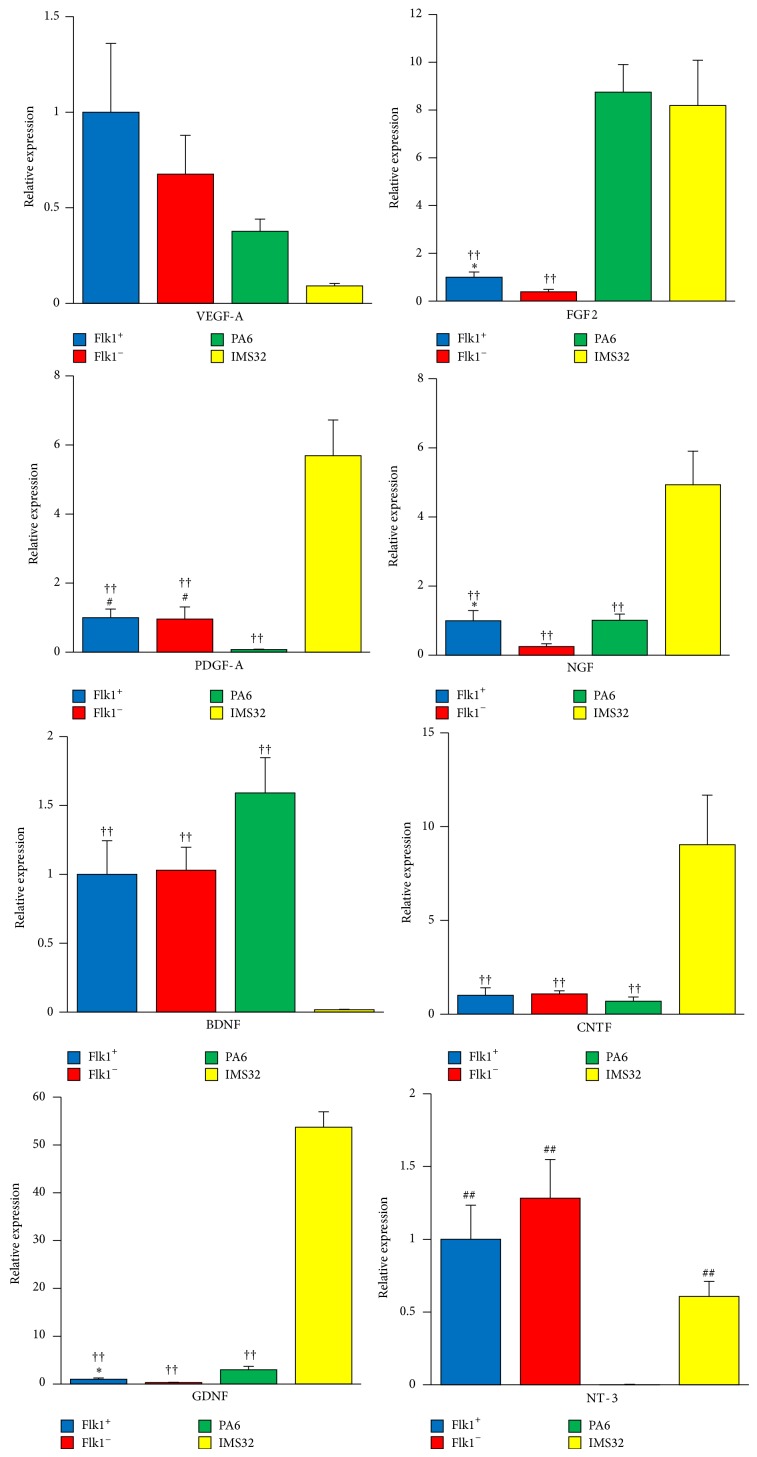
Transcript levels of angiogenic and neurotrophic factors in angioblast-like cells induced from ES cells (ES-ABs). Sorted ES-ABs expressed angiogenic and neurotrophic factors: VEGF-A, PDGF-A, FGF2, NGF, brain-derived neurotrophic factor (BDNF), glial cell line-derived neurotrophic factor (GDNF), Neurotrophin-3 (NT-3), and ciliary neurotrophic factor (CNTF). Each expression level of the factors compared Flk1 positive cells with Flk1 negative cells, PA6 cells, and IMS32 cells. PA6: a cell line of mouse mesenchymal stem cell and IMS32: a cell line of immortalized mouse Schwann cell. Flk1^+^: Flk1 positive cells and Flk1^−^: Flk1 negative cells. ^∗^ *P* < 0.05 versus Flk1^−^ cells, ^##^ *P* < 0.005 versus PA6 cells, and ^††^ *P* < 0.005 versus IMS32 cells.

RT-PCR analyses revealed that sorted angioblast-like cells induced from ES (hereafter referred to as ES-AB) expressed angiogenic and neurotrophic factors: VEGF-A, PDGF-A, FGF2, NGF, brain-derived neurotrophic factor (BDNF), glial cell line-derived neurotrophic factor (GDNF), Neurotrophin-3 (NT-3), and ciliary neurotrophic factor (CNTF) (Figure 2). Expression of FGF2, NGF, and GDNF was significantly increased in ES-ABs compared with Flk1^−^ cells. It is widely known that Schwann cells provide mechanical protection and paracrine effects relying on the production of neurotrophic and angiogenic factors. Therefore, we evaluated the relative expression levels of these growth factors in ES-ABs compared with a mouse Schwann cell line, IMS32. Although the expressions of GDNF, NGF, FGF2, PDGF-A, and CNTF evidenced a significant decrease in ES-ABs compared with IMS32, the expressions of VEGF-A and NT-3 in ES-ABs were comparable to those in IMS32 (Figure 2). Furthermore, the expression of BDNF significantly increased in ES-ABs compared to IMS32. In addition, we compared the relative expression levels of these growth factors between ES-ABs and mouse MSCs, which have been widely applied in ischemic diseases with an expectation of their paracrine effects. Although expression levels of GDNF and FGF2 in ES-ABs showed a significant decrease compared with those in PA6 cells, a cell line of mouse MSCs, there was no significant difference in levels of VEGF-A, NGF, BDNF, and CNTF expressions between ES-ABs and MSCs. Additionally, there were significant increases of NT-3 and PDGF-A expression levels in ES-Abs compared with PA6 cells.

### 3.3. ES-ABs Purified with MACS Differentiated to Endothelial Cells and Smooth Muscle Cells In Vitro

**Figure 3:**
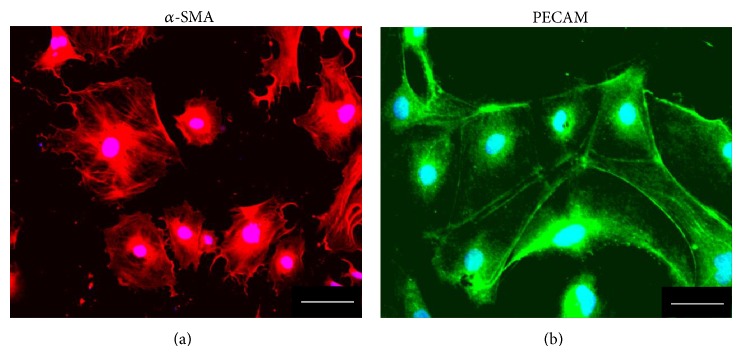
In vitro differentiation potential of angioblast-like cells induced from ES cells (ES-ABs). After a 4-day culture on gelatin, ES-ABs formed many colonies. Immunocytochemistry, a smooth muscle cell marker, *α*-SMA, was detected at some colonies (a), and an endothelial marker, PECAM, was at the other colonies (b). Red: *α*-SMA and green: PECAM. Bar: 100 *μ*m.

Following purification with MACS, the ES-ABs were cultured on gelatin for additional 4 days to assess their abilities to differentiate to endothelial cells and smooth muscle cells. After the culture, cells formed numerous colonies. The use of the immunocytochemistry method revealed that some colonies expressed an endothelial marker, PECAM, and the other colonies expressed a smooth muscle cell marker, *α*-SMA (Figure 3).

### 3.4. Structured Tube Formation and “Cobblestone” Monolayer Were Constructed from ES-ABs

**Figure 4:**
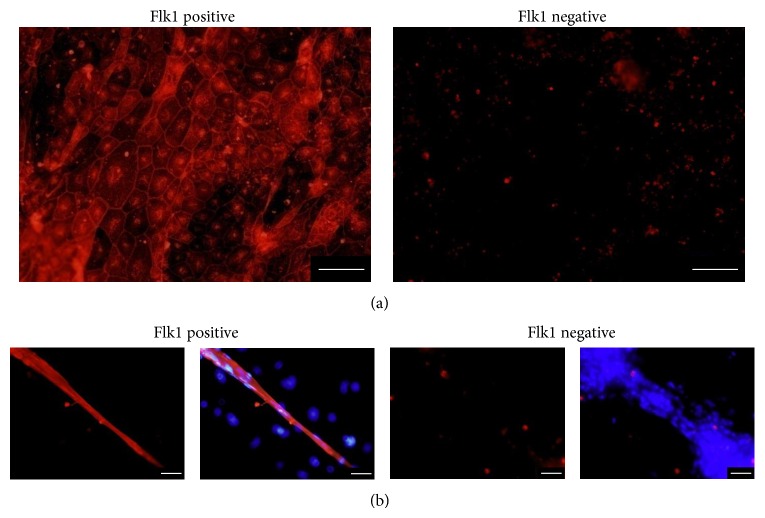
Tube forming assay of angioblast-like cells induced from ES cells (ES-ABs) on Matrigel. To elucidate whether ES-ABs themselves are enabled to form blood vessels, sorted ES-ABs and Flk1 negative cells were, respectively, seeded on Matrigel. Two days later, cells were visualized with isolectin IB4, a stain of endothelial cell. (a) Although endothelial cells stained with IB4 were rarely induced from Flk1 negative cells (right), heavy numbers of endothelial cells were induced from ES-ABs and deployed cobblestone-like pattern (left). Bar: 100 *μ*m. Red: isolectin IB4. (b) In ES-AB culture, a part of cells stained with IB4 structured vessel like tube formation (left 2 images). On the other hand, cells forming a line were not stained with IB4 in culture of Flk1 negative cells (right 2 images). Bar: 100 *μ*m. Red: isolectin IB4 and blue: DAPI.

To elucidate whether ES-ABs themselves are enabled to form blood vessels, sorted ES-AB and Flk1^−^ cells were separately seeded on Matrigel. After culturing for 2 days on Matrigel, cells were visualized with isolectin IB4. Although endothelial cells stained with IB4 were rarely induced from Flk1^−^ cells, significantly heavy numbers of endothelial cells were induced from Flk1^+^ cells. In addition, a portion of these endothelial cells restructured into a vessel-like tube formation, and the remaining portion deployed into a cobblestone-like configuration (Figures 4(a) and 4(b)).

### 3.5. Body Weights, Blood Glucose Levels, and HbA1c

At 12 weeks, diabetic mice showed severe hyperglycemia (random blood glucose levels: *P* = 0.0003. HbA1c: *P* < 0.0001) and significantly decreased body weight gain (*P* = 0.003) (Table 2). After the transplantation of ES-ABs, there was no significant change between transplanted and nontransplanted mice (Table 2).

### 3.6. Some Blood Vessel Walls and Capillaries Were Constructed with Transplanted Cells

**Figure 5:**
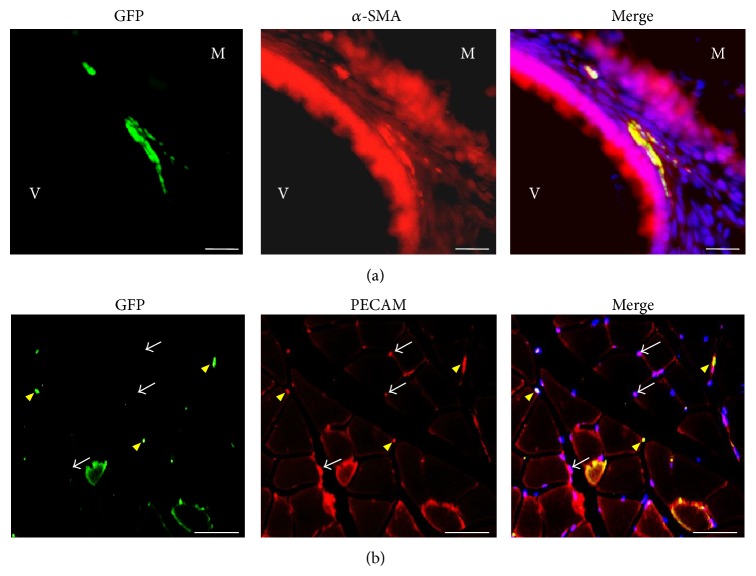
The transplanted angioblast-like cells induced from ES cells (ES-ABs) constructed blood vessel walls and capillaries. Immunohistochemical staining revealed that transplanted GFP-expressing ES-ABs were still engrafted 4 weeks after transplantation. (a) The engrafted cells distributed in vessel wall between hindlimb muscle fibers and coexpressed *α*-SMA. Green: GFP and red: *α*-SMA. M: muscle and V: lumen of a blood vessel. Bar: 20 *μ*m. (b) Some of GFP positive cells resided in gaps between soleus muscle fibers and coexpressed PECAM. Green: GFP and red: PECAM. White arrows: GFP negative capillaries and yellow arrowheads: GFP positive capillaries. Bar: 20 *μ*m.

To detect the distribution of transplanted cells, several mice were injected with GFP-expressing ES-ABs. To determine the existence of teratomas, four weeks after the transplantation, the muscles, brains, hearts, lungs, and livers of these mice were collected and sectioned. GFP positive (GFP^+^) cells were nonexistent except in muscles of the transplanted hindlimbs. GFP^+^ cells resided in the gaps between muscle fibers and some GFP^+^ cells were observed in the walls of blood vessels interspersed with GFP negative cells (Figure 5). Some of the GFP^+^ cells found within the construct of the vessel walls were smooth muscle cells expressing *α*-SMA (Figure 5(a)), while the others were endothelial cells expressing PECAM (Figure 5(b)).

### 3.7. Capillary Number to Muscle Fiber Ratio

**Figure 6:**
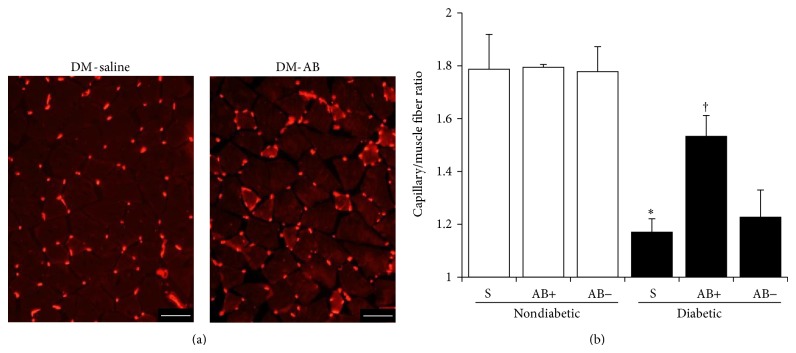
Capillary number to muscle fiber ratios in soleus muscles. (a) The vasculatures were visualized by Alexa594-conjugated isolectin IB4 in soleus muscles. More capillaries existed in soleus muscle of transplanted diabetic mice (right) compared to those of nontransplanted control diabetic mice (left). Red: isolectin IB4. Bar: 50 *μ*m. DM-saline: saline-injected limb in diabetic mice and DM-AB: limbs transplanted angioblast-like cell derived from ES cells in diabetic mice. (b) Quantitative analyses revealed that the capillary number to muscle fiber ratios in the saline-injected sides of diabetic mice were significantly reduced compared with those of normal mice (N). Transplantation of angioblast-like cell derived from ES cells (ES-ABs) significantly augmented the ratios in transplanted limbs compared with those in saline-injected side limbs in diabetic mice (DM). Transplantation of ES-ABs into normal mice showed no significant differences. S: saline treated limbs, AB+: limbs transplanted ES-ABs, and AB−: contralateral limbs transplanted ES-ABs. Results are means ± SD. ^∗^ *P* < 0.05 versus S in N (N-S) and ^†^ *P* < 0.05 versus S in DM (DM-S). *N* = 4 in N-S and *n* = 3 in DM-S (*P* = 0.0004). *N* = 3 of AB+ in DM and *n* = 5 of AB− in DM (*P* = 0.0276 represents DM-S versus AB+ in DM). *N* = 4 of AB+ in N and *n* = 4 of AB− in N (*P* = 0.9609 represents N-S versus AB+ in N).

The vasculatures were visualized by Alexa594-conjugated isolectin IB4, a marker for endothelial cells (Figure 6(a)). Quantitative analyses revealed that the capillary number to muscle fiber ratios in the saline-injected diabetic mice were significantly reduced compared with those in normal mice (*P* = 0.0004) (Figure 6(b)). Transplantation of ES-ABs significantly augmented the ratio in ES-AB transplanted limbs (ES-AB^ipsi^) compared with the ratio in the saline-injected side limbs (ES-AB^contra^) in diabetic mice (*P* = 0.0276) (Figure 6(b)). Transplantation of ES-ABs into normal mice showed no significant differences (*P* = 0.9609) (Figure 6(b)).

### 3.8. Transplantation of ES-ABs Increased Blood Flow in the Sciatic Nerve and Plantar Skin

**Figure 7:**
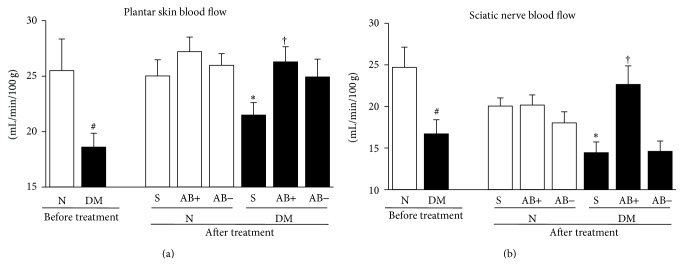
Blood flow of sciatic nerves and plantar skins were ameliorated by transplantation of angioblast-like cell derived from ES cells (ES-ABs). At a time point of 12 weeks of diabetes (before treatment), blood flows of plantar skins (a) and sciatic nerves (b) in diabetic mice significantly decreased compared with those in normal mice, and, 4 weeks after transplantation (after treatment), the decreases were ameliorated in transplanted limbs of diabetic mice. However, administration of ES-ABs did not alter blood flow in those of normal mice. N: normal mice limbs, DM: diabetic mice limbs, S: saline-injected limbs, AB+: limbs transplanted ES-ABs, and AB−: contralateral limbs transplanted ES-ABs. Results are means ± SD. ^#^ *P* < 0.05 versus pretreatment N. ^∗^ *P* < 0.05 versus posttreatment S-treated N, ^†^ *P* < 0.05 versus posttreatment S-treated DM. *n* = 4–6 in each pretreatment group and *n* = 7–10 in each posttreatment group.

After 12 weeks of diabetes, the blood flow in both the sciatic nerve and plantar skin in diabetic mice decreased significantly compared with that in normal mice (sciatic nerve: *P* = 0.0431; plantar skin: *P* = 0.0359), and the decrease was ameliorated by transplantation of ES-ABs (sciatic nerve: *P* = 0.0003 represents DM-S versus DM-ES-AB^ipsi^; plantar skin: *P* = 0.0211 represents DM-S versus DM-ES-AB^ipsi^). However, administration of ES-ABs did not alter the blood flow in normal mice (sciatic nerve: *P* = 0.9525 represents N-S versus N-ES-AB^ipsi^; plantar skin: *P* = 0.2523 represents N-S versus ES-AB^ipsi^) (Figure 7).

### 3.9. Reduced Sensory Perception in Diabetic Mice Was Ameliorated by ES-AB Transplantation

**Figure 8:**
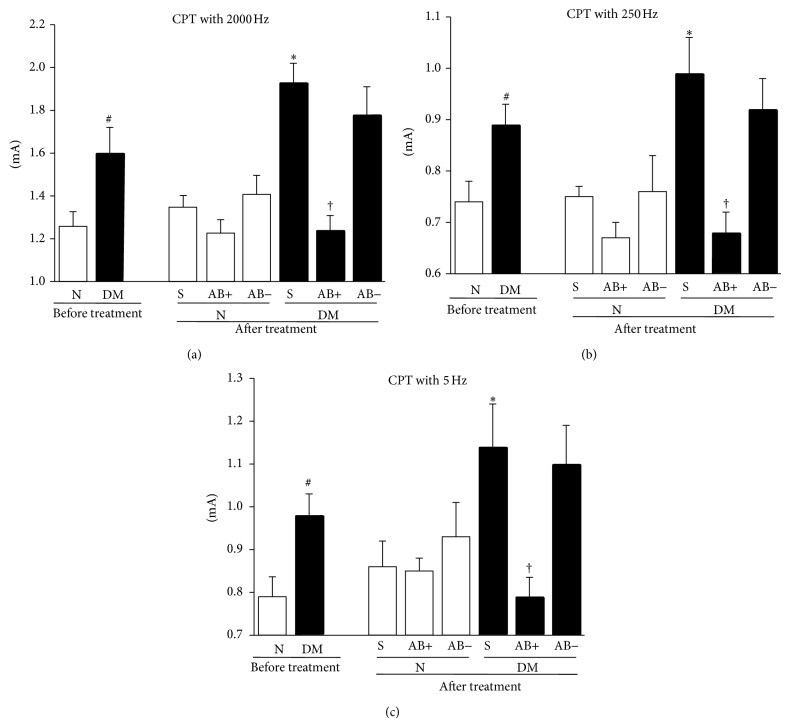
Impaired sensory perceptions in diabetic mice were ameliorated by angioblast-like cells induced from ES cells (ES-ABs) transplantation. Stimuli with frequencies of 5 Hz (a), 250 Hz (b), and 2000 Hz (c) evoked excitations of C-fiber, A*δ*-fiber, and A*β*-fiber, respectively. Before cell transplantation, CPTs with all kinds of stimuli in diabetic mice had significantly increased compared with those in normal mice, representing hypoalgesia in diabetic mice. Four weeks after the transplantation of ES-ABs, these deficits in sensation were significantly improved in diabetic mice compared with saline-treated diabetic controls. The transplantation of ES-ABs into normal mice did not induce significant changes in CPTs. N: normal mice limbs, DM: diabetic mice limbs, S: saline-injected limbs, AB+: limbs transplanted ES-ABs, and AB−: contralateral limbs transplanted ES-ABs. Results are means ± SD. ^#^ *P* < 0.05 versus pretreatment N, ^∗^ *P* < 0.05 versus posttreatment S-treated N, and ^†^ *P* < 0.05 versus posttreatment S-treated DM. *n* = 8–10 in each nondiabetic group and *n* = 7–9 in each diabetic group.

After 12 weeks of diabetes, CPTs at 5, 250, and 2000 Hz had significantly increased compared with those in normal mice (5 Hz: *P* = 0.015; 250 Hz: *P* = 0.019; 2000 Hz: *P* = 0.028), representing hypoalgesia in diabetic mice. The fourth week after the transplantation of ES-ABs, these deficits in sensation had significantly improved in diabetic mice compared with saline-treated diabetic controls (5 Hz: *P* = 0.071 represents DM-S versus DM-ES-AB^ipsi^; 250 Hz: *P* = 0.0018 represents DM-S versus DM-ES-AB^ipsi^; 2000 Hz: *P* < 0.0001 represents DM-S versus DM-ES-AB^ipsi^). The transplantation of ES-ABs into normal mice did not induce significant changes in CPTs (5 Hz: *P* = 0.934 represents N-S versus N-ES-AB^ipsi^; 250 Hz: *P* = 0.212 represents N-S versus N-ES-AB^ipsi^; 2000 Hz: *P* = 0.260 represents N-S versus N-ES-AB^ipsi^) (Figure 8).

### 3.10. ES-ABs Improved Delayed NCVs in Diabetic Mice

**Figure 9:**
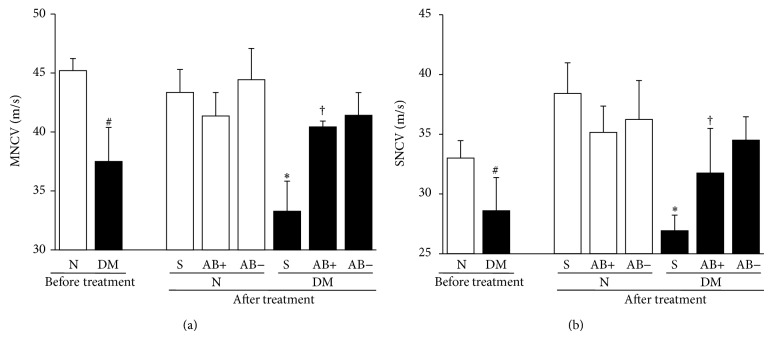
Transplantation of angioblast-like cells induced from ES cells (ES-ABs) improved delayed nerve conduction velocities (NCVs) in diabetic mice. Motor nerve conduction velocity (MNCV) (a) and sensory nerve conduction velocity (SNCV) (b) of diabetic mice were significantly delayed compared with those of normal mice after 12 weeks duration of diabetes. The delays in MNCV and SNCV were significantly restored by ES-ABs transplantation. However, administrations of ES-ABs did not alter NCVs in normal mice. N: normal mice limbs, DM: diabetic mice limbs, S: saline-injected limbs, AB+: limbs transplanted ES-ABs, and AB−: contralateral limbs transplanted ES-ABs. Results are means ± SD. ^#^ *P* < 0.05 versus pretreatment N, ^∗^ *P* < 0.05 versus posttreatment S-treated N, and ^†^ *P* < 0.05 versus posttreatment S-treated DM. *n* = 6 in each pretreatment group and *n* = 7–10 in each posttreatment group.

MNCV and SNCV of diabetic mice were significantly delayed compared with those of normal mice (Figure 9). The delay in MNCV and SNCV was significantly restored by ES-AB transplantation (MNCV: *P* = 0.0289 represents DM-S versus DM-ES-AB^ipsi^; SNCV: *P* = 0.0201 represents DM-S versus DM-ES-AB^ipsi^) (Figure 9). However, administration of ES-ABs did not alter NCVs in normal mice (MNCV: *P* = 0.7604 represents N-S versus ES-AB^ipsi^; SNCV: *P* = 0.6016 represents N-S versus N-ES-AB^ipsi^) (Figure 9).

### 3.11. Nerve Fibers in the Epidermis Were Preserved by ES-ABs

**Figure 10:**
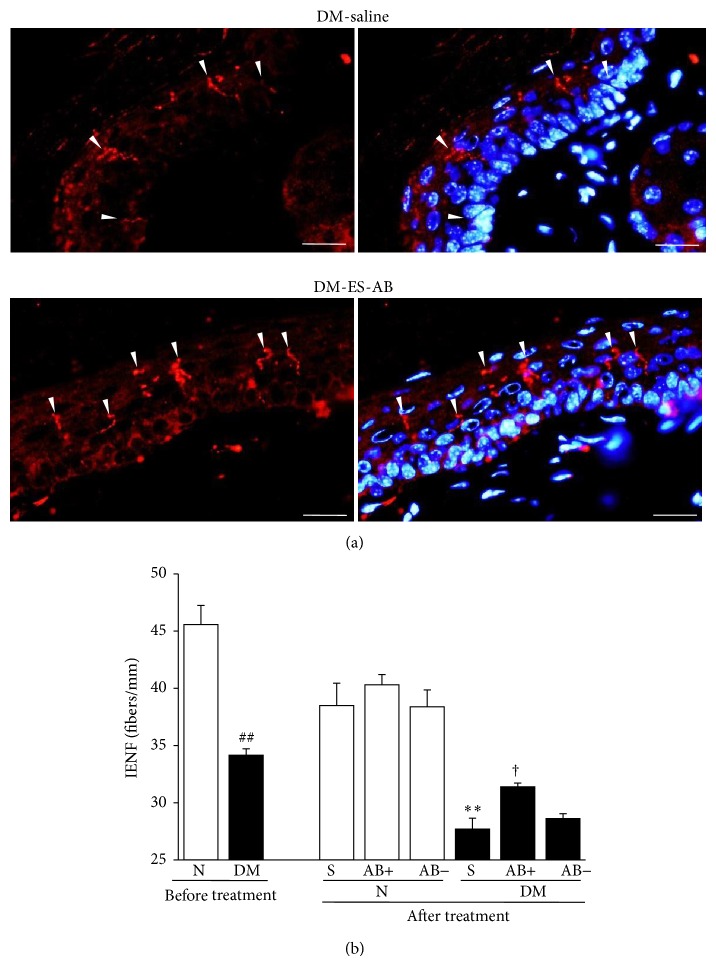
Intraepidermal nerve fibers (IENFs) were preserved by angioblast-like cells induced from ES cells (ES-ABs). (a) IENFs in plantar skins were visualized with PGP9.5 antibody (red). After 15 weeks of diabetes, IENF densities were decreased in diabetic mice (upper) and the impairment was restored by ES-ABs transplantation (lower). DM-saline: plantar skin of saline-injected limbs in diabetic mice and DM-ES-AB: plantar skin of limbs transplanted ES-ABs in diabetic mice. White arrowheads: IENFs. (b) Quantification of IENF densities demonstrated that IENFs were significantly decreased in diabetic mice compared with those in normal mice at 12 weeks of diabetes and this decrease was significantly ameliorated by transplantation of ES-ABs. However, administration of ES-ABs did not change IENF densities in normal mice. N: normal mice limbs, DM: diabetic mice limbs, S: saline-injected limbs, AB+: limbs transplanted ES-ABs, and AB−: contralateral limbs transplanted ES-Abs. Results are means ± SD. ^##^ *P* < 0.005 versus pretreatment N, ^∗∗^ *P* < 0.005 versus posttreatment S-treated N, and ^†^ *P* < 0.05 versus posttreatment S-treated DM. *n* = 3-4 in each group.

Utilization of fluorescent imaging showed that nerve fibers in the plantar skin were evidenced in both the epidermis and the dermis (Figure 10(a)). After 12 weeks of diabetes, IENFDs decreased in diabetic mice (*P* = 0.0011) (Figure 10(b)). This decrease was significantly ameliorated by ES-ABs (*P* = 0.0355 represent DM-S versus DM-ES-AB^ipsi^). Administration of ES-ABs did not change IENFDs in normal mice (*P* = 0.3212 represents N-S versus N-ES-AB^ipsi^) (Figure 10(b)).

## 4. Discussion

The present study demonstrated that angioblast-like cells could be obtained from mouse ES cells and the transplantation of the angioblast-like cells, ES-AB, improved several physiological impairments in DPN: hypoalgesia, delayed NCVs, and reduced blood flow in sciatic nerves and plantar skin. The immunohistological assessment revealed that the capillary number to muscle fiber ratios increased in skeletal muscles of the transplanted hindlimbs, and intraepidermal nerve fiber densities were also ameliorated in the transplanted plantar skin samples. Four weeks after the transplantation, transplanted cells maintained their viabilities and differentiated to endothelial cells and smooth muscle cells around the injected sites. Moreover, several transplanted cells constructed chimeric blood vessels with recipient cells.

One of the most significant characteristics of the current transplantation therapy was the utilization of the heterogeneous graft cells which were at the early stage of differentiation of the pluripotent stem cells. We expected some advantages of a utilization of cells at the early stage, for example, a multipotent differentiation ability, a high migratory capacity, high adaptability to microenvironments of host tissues, a high proliferation ability, and a capacity to produce various growth factors. In previous studies, Flk1^+^ cells derived from mouse pluripotent stem cells have been shown to differentiate into a number of cell types including endothelial cells, smooth muscle cells, and cardiomyocytes in vitro [47, 52]. In the current study, it was demonstrated that the adhesive Flk1^+^ cells derived from mouse ES cells had a multipotent differentiation ability into endothelial cells and smooth muscle cells, similar to the previous studies and expressed several angioblast markers including Flt1, Tie2, and VE-cad. Furthermore, transplanted cells engrafted themselves in gaps between muscle fibers around transplanted sites and were incorporated into the tissue structures of recipient animals. These results indicate that some of the therapeutic effects obtained in this study were mediated through the differentiation of ES-ABs.

Additionally, we demonstrated that ES-ABs secreted a wide array of growth factors, such as, FGF2, VEGF, PDGF, NGF, BDNF, GDNF, NT-3, and CNTF. In previous studies, MSC transplantation has been performed in the field of ischemic diseases, and it has been suggested that its plausible effects would be mediated largely through paracrine actions of locally released arteriogenic cytokines including FGF2 and VEGF [53–55]. Furthermore, FGF2 and VEGF have been reported to have neurosupportive effects [56, 57]. In our study, FGF2, VEGF, and PDGF were expressed in ES-ABs and, in particular, PDGF expression of ES-ABs was significantly higher than that of MSCs. In addition to these conventional angiogenic factors, neurotrophic factors, such as NGF, BDNF, GDNF, NT-3, and CNTF were expressed in ES-ABs. In the peripheral nervous system, these neurotrophic factors are predominantly secreted by Schwann cells. Although NGF and GDNF expressions in ES-ABs were significantly lower than those in Schwann cells, NT-3 and BDNF expressions in ES-ABs were favorably compared with those in Schwann cells. These observations suggest that the interventional effects of ES-AB transplantation on DPN demonstrated in this study would be partially achieved by the paracrine actions of growth factors, and these factors would work as both angiogenic and neurotrophic factors. However, further studies to elucidate which growth factor is relevant to the paracrine action should be considered.

Our study revealed that the capillary number to muscle fiber ratios in hindlimb skeletal muscles were significantly lower in diabetic mice than in normal mice, which is consistent with human studies [58], and the ES-AB transplantation might restore the decrease through induction and construction of neovascularization. The level of therapeutic vasculogenesis prompted by the ES-ABs transplantation was similar to that prompted by EPC transplantation, as we previously reported [44], and the differentiation and incorporation of ES-ABs to vessels were consistent with this report. However, it remains unknown why ES-AB transplantation did not have any effect on normal mice. It can be speculated that diabetic conditions such as hyperglycemia and impaired local microcirculation might cause or require the transplanted ES-ABs to more strongly produce growth factors and promote vasculogenesis. Further studies are in progress in our laboratory.

The impairment of nerve blood flow (NBF) is one of the major pathogenic factors in the development of DPN. Although there are some disagreements as to whether reduced NBF can account for diabetic neuropathy [59, 60], this notion is clinically and experimentally supported by many other studies, which reported that the amelioration of NBF by various treatments improved impaired nerve functions [61–63]. The beneficial effects of ES-AB transplantation on NCVs and NBFs in this study could be comparable to those of the therapeutic modalities reported previously. Reduced capillary number to muscle fiber ratios and blood flows of plantar skin in diabetic mice were ameliorated by transplantation of ES-ABs. Therefore, these ameliorations of NCVs and NBFs may be due in part to vasculogenesis rendered by ES-ABs and, as mentioned above, paracrine effects of angiogenetic and neurotrophic factors produced by ES-ABs. However, well-conceived studies aiming to clarify molecular mechanisms of ES-Abs on each cell type in vascular and nervous systems should be performed in the future. Furthermore, it should be also considered to determine the duration of the beneficial effects using a lengthened intervention by repetitive transplantations.

In the present study, we evaluated sensory nerve functions using the Neurometer CPT/LAB. The Neurometer is a device that measures, selectively, the CPTs in three classes of afferent fibers by applying transcutaneous sine-wave electric stimuli to the skin at three frequencies of 2000, 250, and 5 Hz via surface electrodes at a current intensity in the range of 0.01–9.9 mA. The pulses at 2000, 250, and 5 Hz mainly stimulate large myelinated (A*β*-), small myelinated (A*δ*-), and small unmyelinated (C-) fibers, respectively. The Neurometer is now widely used clinically to evaluate the effects of analgesic drugs and peripheral nerve function in various painful neuropathies including DPN [50, 64–66]. Twelve weeks after the induction of diabetes, diabetic mice showed hypoalgesia in CPTs at all stimuli with differential frequencies, and transplantation of ES-ABs improved these abnormalities. The aforementioned results were consistent with the results regarding the impaired IENFDs restored by the transplantation of ES-ABs in diabetic mice.

In conclusion, we have demonstrated the beneficial effects of transplantation of angioblast-like cells derived from ES cells on DPN. This ES-AB is relatively easy to obtain and can be repeatedly expanded to sufficient numbers for cell therapy. Although further studies designed to reveal other useful aspects of ES-AB transplantation on DPN might be required, transplantation of angioblast-like cells induced from pluripotent cells appears promising as a novel therapeutic strategy for DPN.

## Figures and Tables

**Table 1:** Primer sequences.

Accession number	Gene	Forward primer (5′→ 3′)	Reverse primer (3′→ 5′)
NM_001025250.3	Vegfa	CAGGCTGCTGTAACGATGAA	TTTCTTGCGCTTTCGTTTTT
NM_008006.2	Fgf2	GTGGATGGCGTCCGCGAGAA	ACCGGTTGGCACACACTCCC
NM_008808.3	Pdgfa	GAGATACCCCGGGAGTTGAT	TCTTGCAAACTGCAGGAATG
NM_001048139.1	Bdnf	GCCACCGGGGTGGTGTAAGC	CATGGGTCCGCACACCTGGG
NM_001112698.1	Ngf	GTGAAGATGCTGTGCCTCAA	GCGGCCAGTATAGAAAGCTG
NM_010275.2	Gdnf	CGGACGGGACTCTAAGATGA	CGTCATCAAACTGGTCAGGA
NM_001164034.1	Ntf3	CGAACTCGAGTCCACCTTTC	AGTCTTCCGGCAAACTCCTT
NM_170786.2	Cntf	GCAATCACCTCTGACCCTTC	ACGGTAAGCCTGGAGGTTCT
NM_010612.2	Kdr	GGCGGTGGTGACAGTATCTT	GTCACTGACAGAGGCGATGA
NM_011587.2	Tie1	TCAACTGCAGCTCCAAAATG	TGACAGCTCTGTCCAAAACG
NM_013690.2	Tek	AAGCATGCCCATCTGGTTAC	GTAGGTAGTGGCCACCCAGA
NM_001032378.1	Pecam1	ATGACCCAGCAACATTCACA	AAAACGCTTGGGTGTCATTC
NM_009868.4	Cdh5	ACCGGATGACCAAGTACAGC	TTCTGGTTTTCTGGCAGCTT
NM_010228.3	Flt1	CCAAGGCCTCCATGAAGATA	ATACTGTCAGGGGCTGGTTG
NM_009309.2	T	GGGGTATTCCCAATGGGGGTGGC	GCCAGGCACTCCGAGGCTAGA
NM_011527.2	Tal1	GTCTCTCAGCGAGAGCCGGGA	CGCTCCGTCATCCTGGGGCATA
NM_011355.1	Sfpi1	TCCCATGGTGCCACCCCACA	TTGCTGCCTGTCTCCCCGTG
NM_001142335.1	Lmo2	TGGATGAGGTGCTGCAGATA	GGATGCACAGAGACCATCCT
NM_007393.3	Actb	CATCCGTAAAGACCTCTATGCCAAC	ATGGAGCCACCGATCCACA

**Table 2:** Body weight, blood glucose, and HbA1c levels.

	Normal mice			Diabetic mice		
	Before transplantation	After transplantation		Before transplantation	After transplantation	
		Saline	ES-AB		Saline	ES-AB
HbA1c (%)	4.0 ± 0.1	4.1 ± 0.2	4.1 ± 0.2	7.3 ± 1.4^**^	7.7 ± 2.1^##^	7.7 ± 2.5^##^
Blood glucose (mmol/L)	9.1 ± 1.6	9.2 ± 1.6	9.1 ± 1.6	23.1 ± 2.8^**^	25.8 ± 8.0^##^	24.2 ± 9.0^##^
Body weight (g)	30.6 ± 2.7	31.3 ± 3.0	31.9 ± 3.7	26.1 ± 3.1^**^	25.3 ± 3.2^##^	27.6 ± 1.6^##^

Results are means ± SD. ES-AB: mice transplanted angioblast-like cells induced from embryonic stem cell. ^**^ *P* < 0.005 versus pretreatment normal mice. ^##^ *P* < 0.005 versus posttreatment normal mice.
